# Scarlet Fever

**Published:** 1859-07

**Authors:** H. A. Carrington

**Affiliations:** Hyde Park, Dutchess County, N. Y.


### SCARLET FEVER.

FROM AN ESSAY, BY HENRY A. CARRINGTON, M. D., HYDE PARK, DUTCHESS COUNTY, N. Y.

### SECTION IX.—TREATMENT.

1. *Prophylactic.*—Several remedies have been praised as efficacious in preventing or modifying scarlet fever; the principal one, however, and the only one to which it is necessary, at present, to draw attention, is belladonna. To Hahnemann belongs whatever of credit, or discredit, the proposition for its use may merit. Fancying he had discovered certain effects from the use of the drug, not unlike the certain symptoms of scarlet fever, and ascertaining, as he supposed, that it did cure the disease, and in other cases *prevent* it, he proclaimed to the world another triumph of the great principle, "similia similibus curantur;" asserting that he was able, not only to cure scarlet fever, but to prevent it, and thus to banish it from our systems of nosology, except it should remain as an extinct disease. From that day to this, the remedy has met with very varying fortunes, being lauded on the one part as an infallible preventive, and on the other condemned as utterly worthless. We propose to consider the matter as candidly as possible, uninfluenced by any prejudice against the dogma to which it owes its origin.

The proof on which its partisans must rely is, of necessity, of a very uncertain nature, being entirely negative. That, however, it has no just claims to any such position of universal and infallible efficacy as was claimed for it by Hahnemann is, we believe, established beyond a peradventure. But the respectable character of those who have put forward its claims entitle it a calm and dispassionate investigation; for, notwithstanding its suspicious parentage, it should only be discarded when fairly proved wanting. Here we may say, in passing, that, as in proper doses it can be productive of no harm, those who are not satisfied may safely test the matter for themselves. My own experience in the matter has been limited to a few cases; but in all the instances tried, it failed to exert any protective power, for the children, in every case, took the scarlet fever; nor could I perceive any amelioration in course of the disease.

Some authors have brought forward large statistics to prove its power; and apparently proof enough has been given to set this point at rest, but quite as many authors have brought quite as large an array of figures to prove the opposite; and on this subject we must agree with Dr. Periera, that twenty cases of failure are more conclusive against the opinion, than one thousand of non-concurrence are in favor of it. In this view, the experiments of Dr. Balfour (West. Dis. Chil.) are conclusive against its preservative virtues. He states, "that at the Royal Military Asylum at Chelsea, scarlet fever having broken out, he determined to try the virtues of belladonna. There were 151 boys of whom I had tolerably satisfactory evidence that they had not had scarlatina. I divided them into two sections, taking them alternately from the list, to prevent the imputation of selection. To the first section (76) I gave belladonna; to the second section (75) I gave none. The result was that two in each section were attacked by the disease. The numbers were too small to justify deductions as to the prophylactic power of belladonna; but the observation is good, because it shows how apt we are to be misled by imperfect observation. Had I given the remedy to all the boys, I should probably have attributed to it the cessation of the epidemic." Very few, if any, of those who advocate the claims of the article, have proceeded in the cautious manner of Dr. Balfour, else we are confident we should have heard less of the value of a remedy whose real worth is so purely fictitious. The eccentric or capricious character of scarlet fever is not sufficiently borne in mind; for it is well known that it may, and frequently does, attack only one member of a large family of children; which exemption we refer to the capriciousness of the disease: unless, having a prejudice in favor of belladonna, we have given that, and then we are easily led to reason *post hoc*, etc.

If belladonna had any such power as is claimed for it, any just claim to a position analogous to vaccination, scarlet fever, like small pox, ought by this time to have been very much reduced, both in its frequency and mortality. That no such result has occurred need not here be demonstrated. It may be urged that it has not met with the same general acceptation; but we believe that there are very few physicians who have not at some time made use of the remedy, and who have discarded it on account of its inutility.

That it produces no efflorescence at all corresponding to the scarlatina I am well satisfied, after a sufficient experience. If it ever produces such an effect it must be owing to some idiosyncracy of the subject.

Those who wish to see a full and exhaustive discussion of this subject are referred to an article in the *Brit. and For. Med. Chir. Rev.* for January, 1855.

2. *Hygienic.*—It is important that attention be given to the hygienic conditions in which the patient is placed. The sick room ought, if possible, to be large, and at all events well ventilated. The temperature ought to be kept at a mild and uniform rate. It may be somewhat increased during the latter stages. During the fever, the diet ought to be simple gruel; indeed there is very apt to be a loathing of all solid food, so that only fluids can be given.

As regards the treatment to be pursued subsequent to desquamation, it is safest to confine the patient to the house for at least two weeks after desquamation has taken place. The diet should be mild and nutritious, attention being paid to the function of the bowels and skin; the former being assisted by gentle cathartics, if needed; the latter by warm baths.

3. *Treatment of the eruptive stage.*—In all mild, normal cases, in which the rash comes well out, the fever is not high, and there is no disturbance of the nervous system, the treatment is reduced to its minimum. It is in such cases that it behooves the physician to remember the natural termination we have already alluded to, and to be careful that he does not stand between the patient and his just expectation of recovery. Many special modes of treatment have been vaunted from time to time; at one time it may be emetics, at another bleeding, at another purging, at another the use of some article of the materia medica; but whatever it may be, the fashion thereof soon passeth away. Scarlatina is indeed the result of a specific poison, and a specific remedy *may* be found as certain in its operation as vaccina in variola; it has yet, however, to be discovered, the pretensions of belladonna to the contrary notwithstanding.

In the commencement of the mild cases, a gentle laxative may be administered without harm; but it is not always necessary, nor to be given as a matter of course.

The warm bath is the best means of allaying the heat and dryness of the skin; some suppose that effusion has advantages over the bath; either, however, are efficacious in allaying the febrile movement if it be in excess.

The patient, on removal from the bath, should be wrapped in a blanket and kept warm till in a free perspiration. The bath can be repeated three or four times a day if deemed necessary. The pulse may be safely and effectually controlled by the use of tincture of veratrum virides, in doses of from one to three drops every two or three hours. The special power which the veratrum possesses over the pulse renders its use particularly indicated in this disease; we believe it much better adapted to the treatment of scarlet fever than antimony. It may be administered in combination with diuretics or diaphoretics, or both, so that while soothing the high arterial excitement, the kidneys and the skin may be stimulated to perform their functions.

It sometimes happens that even the milder class of simple or uncomplicated cases present febrile symptoms of great severity. For such cases bleeding has been a sovereign remedy with a great majority of the profession, and is yet with many; but the general and increasing prejudice against bleeding in general has caused many to doubt its efficacy in this disease, and, we believe, with justice. The term *inflammatory*, as applied to this stage of the complaint, has, we are confident, misled many who have thought no farther than the name. The character of the inflammatory action and products ought to convince those who will reflect, that we have a very different type of inflammation from that of peritonitis, or pneumonia, or indeed any of the acute inflammations.

But besides this we have other less dangerous and quite as efficient means of effecting the desired object. Of these, the "wet sheet" or "packing" is one of the most certain and prompt means of arresting the fever, producing free perspiration, and allaying the nervous excitement. The patient being placed in the sheet and warmly covered, is to remain until reaction comes on, when perspiration becoming profuse, he will almost invariably fall into a quiet sleep, and awake much refreshed, with the symptoms every way mitigated. Should the fever return again, the same means may be again resorted to, not forgetting that the free perspiration is a depleting remedy of a powerful nature, and needs to be watched.

Should there be delirium, cold applications to head, sinapisms to the back of the neck and feet, and sometimes leeches to the temples, should be employed. As this delirium is for the most part sympathetic, it will subside without the use of more active measures.

The angina that occurs in cases of simple or normal scarlet fever, requires for the most part no special treatment. It is proper, however, and important that the physician should carefully examine the condition of the fauces at every visit, that he may detect any commencing inflammation of a severe type, as indicated by deep redness and swelling of the mucous membrane, or patches of whitish exudation. To treat this, a weak solution of nitrate of silver,—10 or 15 grains to the ounce of water,—applied three or four times a day; or a solution of sulphate of copper and quinine, 10 grains each to an ounce of rose water, recommended by Drs. Meigs, sen., and J. F. Meigs, and applied as the nitrate of silver, will ordinarily be sufficient. Tincture or infusion of capsicum is highly recommended by many. It does certainly produce very beneficial effects, applied by means of a sponge once or twice a day.

Chlorate of potash, one drachm, dissolved in a quart of water, may be given very freely as a drink. Whether it has any specific action or not, we may be certain it can do no harm.

The mild cases of scarlatina certainly require no more active treatment than that just sketched; many, if not most will as certainly do well without even that. Whatever treatment we may pursue, we must be careful to watch our cases, in order to meet any unfavorable symptoms, as they arise, for while we deprecate any unnecessary medication, we insist upon *promptness* and *decision* in any complications that may arise.

We pass next to the treatment of the more malignant forms.

*Bleeding.*—We are fully convinced that this remedy, if but of small or doubtful value in the treatment of the mild cases, is in this class to be laid aside entirely; at least in any other than a merely topical application, by means of leeches or cupping. We are to remember the caution given in the preceding pages, that it is not any ordinary inflammation we are dealing with; on the contrary, the tendency of scarlatina of this grade, is to the typhoid type—a type of disease, it surely needs no argument to prove, but poorly adapted to bear depletion. The symptoms—delirium, rapid pulse, convulsions, jactitation, muscular twitchings, all indicate the nervous system to be chiefly implicated. Death occurring in this stage, rarely or never shows inflammation of any organ, often not even congestion. *A priori* reasoning would teach us to discard the use of all debilitating measures, and bleeding especially. It is not necessary, however, to resort to such arguments, for we confidently appeal to experience for the confirmation of our teaching. It will be found that the great majority of modern authors agree on this point, regarding blood-letting in any but a very modified form, as being useless, if not positively hurtful. That this is the result of fashion merely, we are not prepared to believe, especially in view of our own experience. Those who still recommend bleeding, urge the necessity of using it in the early stage, and then only with caution. But is it not better to lay aside a remedy of such doubtful virtues, at best; which confessedly, unless used with great caution, is liable to do harm rather than good?

As regards leeching and cupping, inasmuch as the quantity of blood abstracted is generally small, and designed to relieve some local congestion, they may at times be useful. But even here the benefit expected is often not found to result. Besides, the leech bites, sometimes prove very troublesome, being centres of erysipelatous inflammations. In short, we believe that in the great majority of cases the patient *is as well*, if not better, without the use of bleeding in any form, whether topical or general.

*Purgatives.*—All active or violent cathartics we should put under the same condemnation as bleeding, and for the same reason besides this additional argument exists against them, that they must increase the irritation of the bowels, to which we have seen them, is already too strong a tendency.

Active 'cartharsis seems to be even a less rational remedy than bleeding. Purgation cannot eliminate the poison, but it can and will inevitably exhaust the patient, and aggravate some of the worst tendencies of the disease. Indeed, none but the mildest forms of purgatives or laxatives ought to be employed.

*Emetics.*—It may be, as claimed by a large and respectable body of medical men, that emetics in the outset of the attack are beneficial. The disease, however, not unfrequently *takes this matter into its own hands;* for vomiting is, as we have seen, not an unfrequent symptom. A mild emetic, however, in the earliest stage of the complaint is not open to any serious objection; but that it has any such sovereign efficacy as has been claimed for it by some practitioners we do not credit, indeed we know to the contrary. While, therefore, emesis may properly be employed in some cases, and is not likely to be hurtful when used with proper caution, it cannot be relied on, to mitigate the symptoms to any considerable extent. It happens sometimes that large collections of tough, viscid mucus, gather in the throat in young children, who are incapable of removing such matters; in this case a mild emetic of ipecacuanha will be beneficial.

*Chlorate of Potash.*—Within a few years this remedy has come largely into use; and in these cases in which there is a tendency to ulceration of the throat or mouth is a very valuable remedy. Whether it has any specific value, is not yet, we think, ascertained. Dr. Watson recommends a solution of the strength of one drachm to one pint of water, to be given daily, to the extent of one pint or pint and a half; and says, under the use of this solution I have remarked in many instances a speedy improvement of the tongue, which from being furred or brown and dry, has become clean and moist.

*Solution of Chloride of Soda* has not had so general a use, perhaps, as the above; but we believe it to be nearly or quite, equal to it in value. Some physicians who have relied on it, claim quite as satisfactory results as by any other mode of treatment.

*Chlorine.*—Dr. Watson speaks very highly of this article; stating that from several distinct and highly respectable sources, chlorine itself had been urged on his attention, as a most valuable remedy in the severest forms of scarlet fever; his informants stating that whereas they formerly dreaded to be summoned to cases of that disease, they now, having had experience of the virtues of chlorine, felt no misgivings in undertaking the treatment. The formula he gives, is as follows: Put eight grains of the chlorate of potash into a pint bottle, and pour on them one drachm of strong hydrochloric acid; close the bottle until the violent action has ceased, then add one ounce of water and shake the mixture; and so on, until the bottle is full. The chlorate to be pulverized, and in cold weather, the bottle warmed. To be kept in the dark. A table-spoonful or two, according to the age of the patient, was to be given frequently; an adult may take the whole in a day.

Upon the question whether chlorine and its preparations have any specific action in curing scarlet fever, there is not, we conceive, sufficient evidence accumulated to enable us to decide. But this much is certain; there is enough to render them worthy a trial. We shall not stop to discuss the *modus operandi* by which they may be supposed to effect a curative influence; but only to urge the profession to give to these preparations an ample trial before condemning them.

*Tonics and Stimulants.*—We have described certain cases of scarlet fever as being marked from the onset of the attack by symptoms of great nervous prostration, sometimes amounting to collapse. The remedies called for here, are obviously those capable of sustaining the system while suffering under the depressing effect of the poison, and of aiding it in recovering from the injury received; and such remedies are stimulants and tonics.

Of the first class, carbonate of ammonia has been most extensively and, we may add, most extravagantly praised. It is undoubtedly a valuable remedy, and should be freely given from the first appearance of the adynamic symptoms. With it we must give other medicines and stimulants, as wine, brandy, quinine, serpentarian, and capsicum, administered in proportion to the severity of the symptoms. Beef tea should be given freely, to sustain the strength as much as possible, until the crisis has passed.

*Bathing.*—The use of the warm bath in this form of disease, is of even more value than in the milder cases. It quiets the restlessness and agitation, allays delirium, often removes coma and convulsions, and efficiently reduces the heat of the skin. It is true these good effects are often temporary, and sometimes wanting; but in cases at all amenable to treatment, the application of water, whether cool or warm, whether by affusion, sponging or immersion, cannot fail to give great relief. These methods are to be used according to the symptoms, so that no certain directions can be given. Sponging can always be employed, often when bathing is impossible, and may be repeated as often as the heat of the skin returns. The bath may be continued from fifteen minutes to half an hour, and repeated five or six times a day.

*Treatment of the Complication.*

1. *Angina.* The inflammation of the fauces and adjoining parts, as presenting some of the most formidable results of the disease, demand especial attention.

*Leeches* may sometimes be applied with benefit, but in the majority of cases the patient will probably do as well without them; besides the leech bites sometimes inflame and ulcerate, adding mischief to what is already bad enough.

*Cold applications* to the throat externally, in the form either of ice or clothes dipped in ice water, and wrapped around the neck, are found to be of great service sometimes. Dr. Corson strongly recommends that ice be taken in the mouth and allowed to dissolve slowly, a method also commended by Dr. Meigs.

*Cauterization* has been employed with the intention of arresting the inflammation, but the evidence in its favor is at best but doubtful, while by some physicians it is considered as positively hurtful.

Among the most valuable topical applications I believe we must rank cayenne pepper. The powder may be mixed in water and applied to the fauces by means of a small brush. The solution of chloride of soda, diluted with ten parts of water, and used as a gargle, will yield beneficial results.

The collections of viscid mucous in the throat are very annoying, and may prove fatal by stopping respiration. This should be diligently removed, which may be done by a sponge fastened on a stick. It will require to be done every few hours as long as the secretion continues, with the inability to remove it. Astringent washes of alum, or acetate of lead, will aid in removing the mucous and preventing its formation.

The external swellings may be relieved by warm poultices or fomentations. I have seen the application of the tincture of iodine very useful in allaying the inflammation; may be applied two or three times a day.

It happens in some rare cases that croup is developed by the extension of the inflammation to the larynx, and the formation of false membrane. The treatment of such cases cannot be actively antiphlogistic, for the patient will be already too much debilitated by the course of the disease. A solution of nitrate of silver, twenty grains to the ounce, applied by means of a probang and sponge, and even tracheotomy are modes of treatment promising the best results.

*Coryza and Ottorhœa.*—Coryza will be best relieved by cleansing the nasal passage by a weak solution of alum, or sage tea and alum, or by the injection of a mild solution of nitrate of silver. The washes may be applied by means of a small camel's hair brush or sponge.

The ottorrhæa seldom occurs during the violence of the attack, and will not ordinarily require anything more than cleansing the ears two or three times a day, by means of warm water, or warm water and castile soap.

*The retrocession* of the efflorescenee, which sometimes happens, should be treated by means of warm baths, friction with stimulating liniments, and blisters, if necessary. Wilson says that an eruption evincing a disposition to metastasis, may frequently be fixed by means of a blister.

When we have evidence of the *congestion* of internal organs, as the *lungs* or *kidneys*, dry cupping, leeches and blisters, or sinapisms, will greatly suffice to relieve the affected organ.

We have reserved to the present a few remarks upon one or two specialities in the treatment of scarlet fever.

1st. *Cold affusion.*—This mode of treatment was first or mainly brought to the attention of medical men by Dr. Corrie; it has since met with some sanguine supporters, but with the profession at large it has had little acceptance; and it is evident that the conclusions of Dr. Geo. Gregory, are those adopted by the great majority of those who have given it a trial. He says: "Sanctioned by my uncle, the late Dr. Gregory of Edinburgh, this plan has been amply tried in all parts of the world, but it has not realized the expectations of its proposer. The truth is, that the cold affusion is applicable only to a small number of cases. It is adapted for young people with high anginose inflammation and burning hot skin, without plethora, without depression of nervous energy; but it is applicable to the scarlatina of adults, accompanied with coma, phrenitis, or marked debility. It is wholly unfit for cases of cynache maligna. It answers its purpose very well for the first day or two, but it is impossible to continue its use. Lastly, it seems to increase the disposition to dropsy."

We believe that we have in the preceding pages sufficiently indicated the cases requiring, and the best mode of applying water. It is undoubtedly a valuable adjuvant in our treatment of scarlatina, but it does not merit the exalted eulogiums passed upon it. It would certainly be the height of folly and recklessness to employ it in every case. Indeed, Dr. Currie himself relates two cases of malignant scarlatina so treated with a fatal result. In all cases demanding or justifying the use of water, the *warm bath*, *cool or tepid sponging*, and the *wet sheet*, will be found amply sufficient to meet every indication.

2d. *Inunction.*—Dr. Schneemann, of Hanover, first brought to notice the very simple mode of treating scarlatina by inunction with lard. From the first day of the illness, the patient is to be rubbed every morning and evening over the whole body with a piece of bacon, in such a manner that a covering is everywhere applied. The rubbing should be *thoroughly* done, slowly, in order that the skin may be saturated; should be applied twice a day for three weeks, and once a day for the fourth. After that, the patient may be washed with cool water and soap, and not until the skin has become accustomed to the cool solution, should the bath be commenced. The advantages claimed by Dr. S., are shortening of the disease to such an extent that the patient may leave the house at the end of ten days; the checking of all infection by the end of the third or fourth day; the relief of all uneasy and painful feelings in the skin, particularly those that accompany desquamation; the diminution of the amount of desquamation; the prevention of taking cold; and a greater security against complications and sequelæ. The treatment, he observes, is not likely to find much favor with the fastidious, on account of being dirty, but the first few days of its application produce results which make all this to be forgotten, and inspire mothers with enthusiasm. All, even the most painful symptoms, are allayed with rapidity, as if by magic; quiet, sleep, appetite and good humor return, and there remains only impatience to escape the sick room.

Dr. Maushner, of Vienna, gives his testimony in favor of this mode of treatment. Dr. Wilson (Dis. Skin, p. 425), is disposed to think very favorably of it; and Dr. J. F. Meigs speaks of a modification—the use of glycerine—with approbation.

In a few cases in which I have tried this mode as an adjuvant to the ordinary treatment, it did certainly fulfill some of the claims of Dr. Schneemann. But like most other specific modes of treatment, the result is not apt to be so favorable in the hands of others as in that of the inventor or proposer.

The formula given by Dr. Meigs may be employed, probably with quite as beneficial effects as the use of the bacon, and certainly it is far more agreeable and less repulsive. It is as follows:

R. Glycerine—one drachm.
Ungt. aq. Rosæ—one ounce, m.

It will remove the heat and dryness of the skin, the burning and irritation that are so very uncomfortable; and if it did no more, this would be ample reason for employing it. It need not interfere with other appropriate treatment.

3d. *Diluted acetic acid*, proposed by Dr. J. B. Brown, of London. Dr. Brown, besides a course of local and general treatment, well calculated to give relief to the symptoms, adds to that the use of diluted acetic acid: ℞. Distilled vinegar, diluted, one oz., (one part vinegar to seven of water,) syrup f. four drachms, distilled water f. four ounces, m. Two table spoonsful every four hours for a child nine years of age. To be given throughout the entire duration of the disease, of whatever type, and for one or two weeks afterwards, or until desquamation is over.

Dr. Schnect, (*Am. Med. Jour.*, July '57,) has borne very strong testimony to the virtues of this mode of treatment, but I think an examination of his cases will convince any one that his general and local treatment would prevent one from forming an opinion of the efficacy of the acetic acid. The diluted vinegar would form a pleasant drink, and thus recommend itself, even if no specific virtues should be found to belong to the acid.

4th. *Belladonna.*—We have shown already that no virtues appertain to this doing as a prophylactic, and as a specific mode of treatment for the developed disease, we can entertain no more confidence in it; and the great mass of testimony from physicians is concurrent to this effect.

But we hasten to the consideration of the treatment of that most frequent and important sequelæ.

*Dropsy.*—As in many other cases so in the present, an "ounce of prevention is worth a pound of cure." It is of the highest importance, after the acute attack of scarlet fever is past, to protect the patient from all exposures to cold and damp atmospheres. Confinement to rooms of an equable temperature of 68° or 70°, for three weeks, at least, ought to be enforced. Flannel should be worn next to the skin, on which friction with a course towel should be made daily, with the use also of warm baths. The bowels should be kept in a regular and healthy condition. The diet should be farinaceous, with milk, eggs, and occasionally light broths. When these conditions are strictly enforced, the patient is kept in the most favorable condition to avoid the dropsy.

Occasionally dropsy comes on in a very mild form, requiring but gentle treatment; as the warm bath; purgatives, either calomel, or jalap and cream of tartar, in combination; a justly favorite prescription, spirits nitre, digitalis and squills, or tincture of sesqui-chloride of iron.

For another form, attended with febrile symptoms, scanty urine or hæmaturia, a more decided course is required. Most authorities agree in recommending the employment of bleeding as a first and most important measure. We are as much disinclined to the use of the remedy in the sequelæ as in the primary disease, believing that the cases requiring it are very few, and that depletion, when practiced, need be only local, or at least chiefly so.

Where the kidneys are congested, leeching or cupping over the loins to the amount of from two to six ounces, according to the age of the patient, and repeated, if necessary, will be found to relieve the local difficulty as effectually as if larger quantities of blood were taken from the arm, besides we do not, to any extent, weaken the powers of a system already impaired by previous disease.

The next point is to restore the function of the skin; the warm bath, containing common soda, succeeded by friction, should be frequently employed; the exhibition of some of the preparations of antimony along with the warm bath, will materially assist the determination of the skin. The wet sheet is a powerful means of exciting the most profuse diaphoresis; it is a remedy regarded with suspicion by the majority of medical men, but I believe it to be as safe as any other powerful remedy, if used with discretion, and what remedy is safe if used recklessly?

Purgatives, especially hydragogue, are a most efficient means of reducing the effusion. Full doses of the compound jalap powder is one of our most valuable combinations; it should be given every morning; calomel must not be neglected, but can be given in small doses during the day.

When hæmaturia is present, the tincture of sesqui-chloride of iron is strongly indicated, for while producing astringent effects on the capillaries of the kidneys, it assists materially in restoring the blood to its normal state, the blood being always more or less anæmic. The use of the tannic and gallic acids has been recommended by some authors, but we are confident that the tincture of iron will be found capable of producing all the good effects to be expected from them, besides being recommended by its peculiar effects on the blood.

Diuretics, apparently very much needed, must be used with caution, lest we increase the already congested state of the kidneys. Saline diuretics are prohibited during the continuance of the hæmaturia; otherwise they are useful. The preparations of potash, with squills, nitre, or broom, are valuable diuretics.

When the brain is implicated by uræmic poisoning, depletion, general or local, active catharsis, the warm baths and counter irritation, with diuretics, are to be used.

Effusion into the pericardium is sometimes excessive, producing the most distressing symptoms. To meet this it has been mooted whether we ought not to employ the trochar and draw off the fluid. I am not aware that this suggestion has actually been put in practice. The employment of the hydragogue cathartics, as jalap, elaterium, etc., will ordinarily give relief to patients laboring under effusion into the chest, as well as into the cellular tissue and abdomen.

The other sequelæ, as chronic diarrhœa, abscesses, sloughing, erysipelas, require no different treatment from that bestowed upon them as idiopathic or primary affections.

